# SEPIA: simulation-based evaluation of prioritization algorithms

**DOI:** 10.1186/s12911-021-01536-4

**Published:** 2021-06-03

**Authors:** Kimberly Almaraz, Tyler Jang, McKenna Lewis, Titan Ngo, Miranda Song, Niema Moshiri

**Affiliations:** Department of Computer Science and Engineering, University of California San Diego, 9500 Gilman Drive, La Jolla, CA 92093 USA

**Keywords:** SEPIA, HIV, Prioritization, Metrics, Simulation-based evaluation, FAVITES, Phylogenetic

## Abstract

**Background:**

The ability to prioritize people living with HIV (PLWH) by risk of future transmissions could aid public health officials in optimizing epidemiological intervention. While methods exist to perform such prioritization based on molecular data, their effectiveness and accuracy are poorly understood, and it is unclear how one can directly compare the accuracy of different methods. We introduce SEPIA (Simulation-based Evaluation of PrIoritization Algorithms), a novel simulation-based framework for determining the effectiveness of prioritization algorithms. SEPIA expands upon prior related work by defining novel metrics of effectiveness with which to compare prioritization techniques, as well as by creating a simulation-based tool with which to perform such effectiveness comparisons. Under several metrics of effectiveness that we propose, we compare two existing prioritization approaches: one phylogenetic (ProACT) and one distance-based (growth of HIV-TRACE transmission clusters).

**Results:**

Using all proposed metrics, ProACT consistently slightly outperformed the transmission cluster growth approach. However, both methods consistently performed just marginally better than random, suggesting that there is significant room for improvement in prioritization tools.

**Conclusion:**

We hope that, by providing ways to quantify the effectiveness of prioritization methods in simulation, SEPIA will aid researchers in developing novel risk prioritization tools for PLWH.

## Background

Molecular data gathered on human immunodeficiency virus (HIV) is useful for understanding the systems of epidemic spread of HIV. Such understanding can better allow us to intervene and treat high-risk groups of individuals. Methods of epidemic intervention include treatments such as antiretroviral therapy (ART) and awareness programs [1]. Adherence to ART can cause viral suppression in people living with HIV (PLWH) and significantly reduces their risk of transmission, making ART distribution a potentially effective approach to combating the spread of HIV. However, a major issue for public health officials is how to allocate the limited amount of available resources.

In many parts of the world, when testing and treating PLWH, it has become standard practice to record various metadata on the patients, including viral genomic sequences (often of the *pol* and *gag* regions). This information is often used to determine groups of individuals with high risk of future transmission, which can help public health officials better allocate limited resources [2]. The prioritization of PLWH can be explored through a computational framework: given a list of individuals along with metadata and viral sequences, order the individuals in descending order of inferred risk of future transmission.

Molecular epidemiology provides a natural framework for prioritizing individuals from viral sequence data. Currently, the standard approach is to use HIV-TRACE [3] to infer transmission clusters based on pairwise distances between sequences, monitor the growth of the transmission clusters over time, and prioritize individuals in descending order of transmission cluster growth. ProACT [4], on the other hand, is a prioritization approach that utilizes properties of a phylogeny inferred from the viral sequences.

The following questions naturally arise: how well does a given prioritization method perform, and which method is superior in specific contexts? With real-world data, the ground truth of who transmitted to whom is typically unavailable or error-prone. Further, even *with* a known transmission history, it is unclear how to quantify effectiveness: do we count the number of transmissions from a single individual, or the total number of transmissions in a transmission chain seeded from a single individual, or perhaps we are interested in properties of the underlying contact network (e.g. individuals with large numbers of social contacts)? Thus, it is unclear how to quantitatively assess the performance of different prioritization methods.

To address this open problem, we introduce SEPIA (Simulation-based Evaluation of PrIoritization Algorithms), a novel simulation-based framework for measuring the effectiveness of prioritization algorithms. Previously, in Moshiri et al. (2021) [4], ProACT and HIV-TRACE were compared with respect to effectiveness, but the comparisons were limited to a simulated epidemic dataset modeling the San Diego HIV epidemic between 2005 and 2014. Like this prior work, SEPIA utilizes simulated epidemic data, such as those generated by FAVITES [5] or PANGEA.HIV.sim [6], to define a ground truth with which prioritization methods can be directly compared. However, SEPIA expands upon this prior work by generalizing the task of prioritization effectiveness comparison and further exploring the mathematical meaning of “effectiveness” by defining 6 metrics of effectiveness, each inspired by properties of epidemics that are inherently of interest to public health officials for intervention. Specifically, the user runs a prioritization method on a simulated dataset; then, given the prioritization and the simulated dataset, SEPIA will measure the effectiveness of the prioritization using the metrics defined below.

## Implementation

**Fig. 1:**
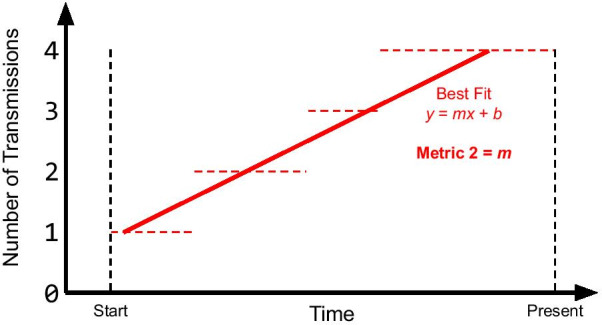
Metric 2 is the slope of the best-fit line (red solid line) of the step function of the number of times a given individual has transmitted (red dashed lines), regressed between the time of the individual’s first transmission event (“Start”) and present day (“Present”)

Given a prioritization, SEPIA computes an effectiveness score according to one of the following metrics:

- **Metric 1: Direct Transmissions** This metric aims to quantify the direct impact of each individual *u* on the spread of the virus within a population by counting the total number of individuals to whom *u* directly transmitted.
- **Metric 2: Transmission Rate** This metric aims to quantify the rate of transmission of each individual *u*, giving higher values to those who transmitted most frequently and most recently. We produce a step function representing the number of transmissions from individual *u* over time, and we measure the slope of a regression line inferred from the step graph (Fig. 1).
- **Metric 3: Indirect Transmissions** This metric expands on Metric 1 to quantify an individual’s broader impact on the community. For an individual *u*, we count the number of individuals who were infected by somebody who was infected by *u*.
- **Metric 4: Direct and Indirect Transmissions** This is the sum of Metrics 1 and 3.
- **Metric 5: Number of Contacts** This metric measures each individual’s total number of contacts in the underlying contact network.
- **Metric 6: Number of Contacts and Transmissions** This is the sum of Metrics 1 and 5.

**Fig. 2:**
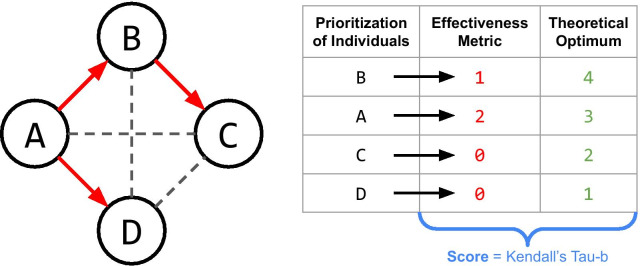
Given simulated epidemic data and a prioritization of the individuals in the simulated epidemic, SEPIA computes the user-selected effectiveness metric for each person in the prioritization. Then, to construct an overall effectiveness score for this prioritization, SEPIA computes the Kendall Tau-b correlation coefficient between the ordered list of effectiveness values and the theoretical optimum

Given a prioritization of *n* individuals and the simulated data from which the prioritization was produced, for a given selected metric, SEPIA will compute a value for each individual in the prioritization. To compute a score comparing the user’s prioritization to the theoretical optimum, SEPIA computes the Kendall Tau-b rank correlation coefficient [7] between the list of ordered metric values and the descending list of integers from *n* to 1 (Fig. 2). The resulting Tau-b score ranges from 1 (perfectly correlated with optimal ordering) to 0 (no better than random ordering) to -1 (perfectly anticorrelated with optimal ordering).

We used SEPIA to compare the effectiveness of two molecular epidemiological prioritization methods. One approach is to use HIV-TRACE to infer transmission clusters from pairwise distances of viral sequences, monitor the growth of the transmission clusters over time, and then to prioritize individuals in descending order of transmission cluster growth. The other approach is ProACT [4], a method that utilizes properties of a phylogeny inferred from the viral sequences. We used a simulated dataset produced by FAVITES to emulate the HIV pandemic in San Diego between 2005 and 2014 [5]. The simulated datasets vary the expected degree in the contact network $\left( E_d\right)$, the rate at which individuals begin ART $\left( \lambda _+\right)$, and the rate at which individuals stop adhering to ART $\left( \lambda _-\right)$.

## Results

**Fig. 3:**
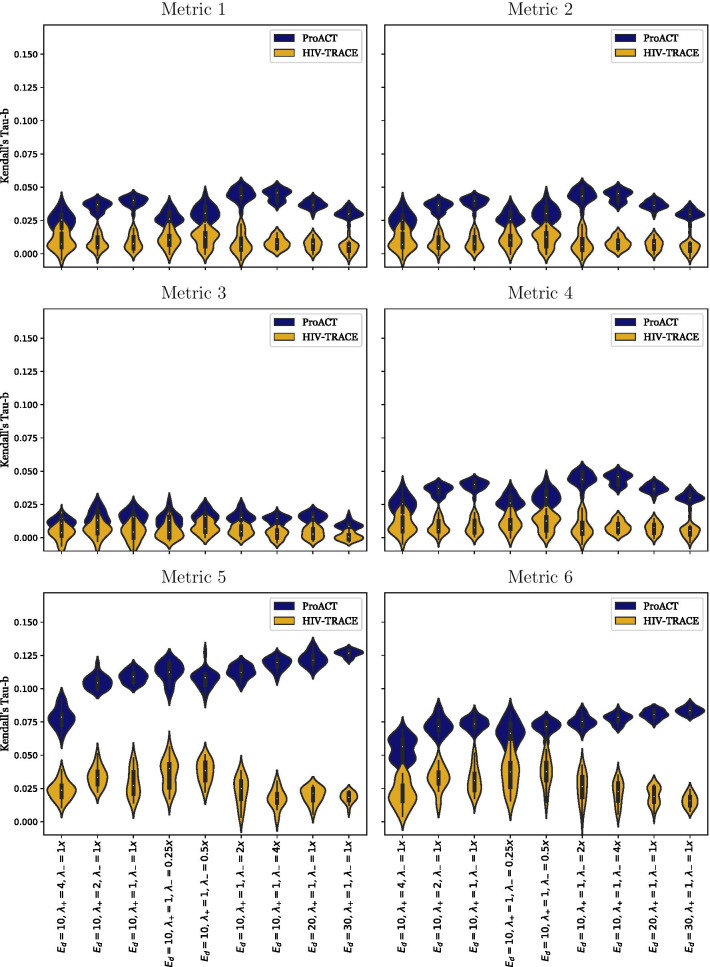
Effectiveness of prioritization using ProACT and HIV-TRACE transmission cluster growth across all metrics on datasets simulated by FAVITES. Each column represents a single experimental condition, and each violin plot depicts the Kendall Tau-b correlation coefficients computed by SEPIA across 20 simulation replicates. The experimental conditions are varied by altering 3 parameters: expected number of contacts per individual $\left( E_d\right)$, rate of starting ART $\left( \lambda _+\right)$, and rate of stopping ART $\left( \lambda _-\right)$

As can be seen in Fig. 3, ProACT consistently outperformed HIV-TRACE transmission cluster growth using all metrics on all simulation conditions. However, both tools consistently had Tau-b scores marginally higher than 0, implying that they are performing only marginally better than a random ordering. As the rate of starting ART $\left( \lambda _+\right)$ increases, the rate of stopping ART $\left( \lambda _-\right)$ increases, and the expected degree $\left( E_d\right)$ increases (i.e., as the outbreak spreads more quickly), ProACT’s performance with respect to metrics 5 and 6 seems to increase slightly. Otherwise, both ProACT and HIV-TRACE transmission cluster growth perform consistently across experimental conditions.

## Discussion

Across all defined metrics and all considered simulation conditions, ProACT consistently outperformed prioritization by HIV-TRACE transmission cluster growth. However, both approaches consistently performed just marginally better than a random ordering, implying that there is room for significant improvement in the realm of HIV prioritization.

## Conclusions

It must be noted that, while we aimed to provide generalized results by varying key simulation parameters, a key limitation of this study is that the simulated epidemics are specifically modeled after the HIV epidemic in San Diego between 2005 and 2014. In practice, molecular epidemiologists will need to assess prioritization techniques using simulated datasets representative of the pathogens and communities in which they are specifically interested.

Further, the 6 metrics we have implemented are by no means exhaustive, but rather, they are simply natural metrics of interest to public health officials. SEPIA is expandable, and we leave the implementation of novel metrics of effectiveness for future works.

We hope that SEPIA will enable researchers to quantify and assess the effectiveness of different prioritization approaches in order to select the best existing prioritization method for their communities, develop new prioritization methods that improve upon existing ones, and, ultimately, maximize the impact of the limited resources available to public health officials.

## Availability and requirements

- Project name: SEPIA
- Project home page: https://github.com/Niema-Lab/SEPIA
- Operating system(s): Platform independent
- Programming language: Python
- Other requirements: SciPy
- License: GNU GPL v3.0
- Any restrictions to use by non-academics: Contact authors
- Manuscript data: https://github.com/Niema-Lab/SEPIA-paper-final

## Abbreviations

ART: Antiretroviral therapy
$$E_d$$: Expected degree
HIV: Human immunodeficiency virus
PLWH: People living with HIV
SEPIA: Simulation-based Evaluation of PrIoritization Algorithms
$\lambda _+$: Rate of starting ART
$\lambda _-$: Rate of stopping ART
